# Evaluating the reliability, validity and minimally important difference of the Taiwanese version of the diabetes quality of life (DQOL) measurement

**DOI:** 10.1186/1477-7525-6-87

**Published:** 2008-10-28

**Authors:** I-Chan Huang, Jung-Hua Liu, Albert W Wu, Ming-Yen Wu, Walter Leite, Chyng-Chuang Hwang

**Affiliations:** 1 Department of Epidemiology and Health Policy Research, College of Medicine, University of Florida, Gainesville, FL, USA; 2 Department of Health Policy and Management, Bloomberg School of Public Health, Johns Hopkins University, Baltimore, MD, USA; 3 Department of Medicine, School of Medicine, Johns Hopkins University, Baltimore, MD, USA; 4 Tainan Hospital, Department of Health, Tainan, Taiwan; 5 Department of Educational Psychology, College of Education, University of Florida, Gainesville, FL, USA

## Abstract

**Background:**

Few diabetes HRQOL instruments are available in Chinese language. We tested psychometric properties of a Diabetes Quality of Life (DQOL) in Chinese language for diabetes patients in Taiwan and estimated its minimally important differences (MIDs).

**Methods:**

Data were collected from 337 patients treated in diabetes clinics of a Taiwan teaching hospital. Pearson's correlations among domain scores of the DQOL (satisfaction, impact, and worry), the D-39S (a diabetes-specific instrument, including domains of diabetes control, energy and mobility, social burden and anxiety and worry, and sexual functioning) and the RAND-12 (a generic instrument, including physical health composite (PHC) and mental health composite (MHC)) were estimated to determine convergent/discriminant validity. Known-groups validity was examined using 2-hour postprandial plasma glucose (2 h PPG), hemoglobin A1c (HbA1c)) and presence of complications (retinopathy, neuropathy, and diabetic foot complications rather than the known groups of cardiovascular and cerebrovascular complications). We used a combined anchor- and distribution-based approach to establish MIDs.

**Results:**

The DQOL scores were more strongly correlated with the physical domains of the D-39S (diabetes control and energy and mobility) and RAND-12 PHC than psychological domains of the D-39S (social burden, anxiety and worry, and sexual functioning) and RAND-12 MHC. The DQOL showed satisfactory discriminative ability for the known groups of 2 h PPG and HbA1c (effect size (ES) ≥ 0.2) and retinopathy, neuropathy, and diabetic foot complications (ES ≥ 0.3), but less satisfactory for the known groups of cardiovascular and cerebrovascular complications. MIDs for the DQOL domains were 3–5 points for satisfaction, 4–5 points for impact, 6–8 points for worry, and 3–4 points for overall HRQOL.

**Conclusion:**

We validated a DQOL in Chinese language for diabetes patients in Taiwan and provided MIDs to facilitate the measure of diabetes HRQOL.

## Background

Diabetes mellitus (DM) is associated with long-term damage of multiple organ systems and increased age-adjusted mortality rates. Conventional assessment for diabetic patients relies on clinical measures, e.g., glycemic control and diabetes complications. However, the use of clinical measures alone for diabetes management is limited because clinical measures can not fully capture patient's health outcomes, especially psychological impact [1]. Health-related quality of life (HRQOL) measures, which emphasize daily functioning and well-being, are useful adjuncts to clinical indicators for assessing diabetic health outcomes.

Several instruments are available for assessing diabetes HRQOL, including generic and diabetes-specific instruments. Generic instruments measure HRQOL domains which are universally important across diseases, while diabetes-specific instruments measure specific impacts of diabetes on functioning and well-being. Specific instruments may be more sensitive to patients' score changes over time [2,3].

There is a great need to develop and validate diabetes HRQOL instruments for Chinese populations, which comprise the largest group of people with diabetes [4]. Although more than a dozen of diabetes HRQOL instruments have been developed [5,6], only three instruments are available in Chinese language (including a translated Diabetes-39 (D-39) [7] and a translated Diabetes Impact Measurement Scales (DIMS) [8] for Chinese people in Taiwan, and a translated Diabetes Quality of Life (DQOL) [9] for Chinese people in Canada). Each instrument, however, may measure somewhat different concepts of HRQOL. For example, the D-39 measures the concepts of physical functioning and psychosocial well-being associated with diabetes including the domains of energy and mobility, diabetes control, anxiety and worry, social burden, and sexual functioning [10], whereas DQOL measures the burden associated with diabetes treatment and glycemic control including the domains of satisfaction with treatment, impact of treatment, and worry about future effect of diabetes [11]. Therefore, it is important to validate and compare different instruments within the same population and to test whether one instrument may be used combined with another to better capture comprehensive diabetes HRQOL.

In testing the usefulness of diabetes HRQOL instruments, the selection of psychometric methods and clinical variables can influence the success of instrument validation [7]. Hemoglobin A1c (HbA1c) – a measure reflecting a longer-term glycemic control – is commonly used as an external variable to validate instruments, but the association between HbA1c and HRQOL is weak [12]. Validation might be improved by further including other laboratory indicators (e.g., fasting plasma glucose (FPG) and postprandial plasma glucose (PPG)) to better account for the impact of fluctuations and acute increase of glycemia (hyperglycemic spikes) on health [13,14]. Additionally, hyperglycemic symptoms and diabetic complications are major determinants of HRQOL [15,16]. The use of laboratory indicators (FPG, PPG and HbA1c) together with diabetes complications would be helpful for validating diabetes HRQOL instruments.

An issue limiting the use of diabetes HRQOL measures is that little guidance is available to interpret HRQOL scores, especially clinical meaning in score difference among treatment groups or score change within individuals over times [5,17]. Conventionally, the interpretation of score changes/differences relies on tests of statistical significance. Yet, statistical significance is not equivalent to clinical significance because the former does not directly link to clinical sensibility and is partially determined by sample size [3]. Clinicians are interested in interpreting score differences, especially minimally important difference (MID) which can serve as the lowest benchmark to determine clinical meaning of HRQOL scores [3,18].

Two methods are commonly used to determine MID: distribution-based and anchor-based approaches [3,19]. Distribution-based approaches rely on statistical properties of the sample (e.g., variation of score distribution) or the instrument (e.g., measurement precision of scale) to establish clinically meaningful change [19]. Anchor-based approaches assess the extent to which changes in measurement instruments correspond to a minimally important change defined by external indicators. These indicators may include clinical variables (e.g., laboratory and physiological measures and clinical ratings) and patient-reported outcomes (PRO) (e.g., global change in health) [20].

To date, there is no consensus on the best approach to evaluate MID [3,19]. Studies have recommended that MID estimations should apply anchored-based approaches using clinical and/or PRO indicators combined with supportive information from the distribution-based estimates to generate a small range of values for MID [20-23]. The strength of using multiple approaches to establish a range of MID is to demonstrate variability among estimates.

The main purpose of this study was to validate and interpret a Taiwanese version of the DQOL [24]. We evaluated the psychometric properties of the DQOL using several clinical variables: 1) laboratory indicators: fasting plasma glucose, 2-hour postprandial plasma glucose, and HbA1c, and 2) complications of diabetes: retinopathy, neuropathy, diabetic foot disorder, cardiovascular, and cerebrovascular diseases. To better interpret the DQOL, we estimated the MID using a combined anchor-based and distribution-based approach.

## Methods

### Participants and data collection

Data were collected from the Taiwan Diabetes Health Survey, an initiative to systematically develop HRQOL instruments for diabetes patients. In the second-year of the project, we focused on the DQOL and assessed its psychometric properties. Face-to-face interviews were conducted by two trained research associates for type-1 and type-2 diabetes patients who utilized outpatient services in the Tainan Hospital – a Taiwan's Department of Health (DOH) affiliated teaching hospital – between 07/2006–10/2006. In total, data from 337 diabetes patients were collected for the statistical analysis. This study was approved by the Institutional Review Board of the Tainan Hospital and received informed consent from each patient.

Data on laboratory measure, clinical diagnosis and HRQOL assessment were collected at the same time from individual patients and tested using the same methods for all patients. Laboratory indicators include fasting plasma glucose (FPG), 2-hour postprandial plasma glucose (2 h PPG), and HbA1c. Diabetes complications were abstracted from medical records, including retinopathy (none vs. background, proliferative, or decreased vision), neuropathy (none vs. present), diabetic foot disorders (none vs. foot ulceration, sepsis, or amputation), cardiovascular complications (none vs. angina, or previous myocardial infarction or congestive heart failure), and cerebrovascular complications (none vs. transient ischemic attack, or stroke).

### Background of developing the DQOL

The DQOL was originally developed to assess HRQOL for type-1 diabetes [11] and has been adapted for type-2 diabetes [25-27]. The original DQOL consists of 46 items measuring the domains of satisfaction with treatment, impact of treatment, worry about future effects of diabetes, and worry about social/vocational issues [11]. The DQOL has been translated to Chinese language for people in Canada [9], with a modification of the original instrument (i.e., adding and replacing some items) to capture culture-sensitive issues such as eating and sexual activities. These modifications are necessary because eating style and joyfulness are essential components of Chinese culture where family gathering and social activities are centered on meals. By contrast, sexual activity is a taboo subject in Chinese culture especially among elderly people who are less willing to reported sexual functioning. Our previous study suggests that measuring sexual functioning by diabetes elderly people is less reliable and less valid compared to other diabetes HRQOL domains [7].

The extant Chinese version developed for Chinese people in Canada can not be directly applied to our study population because different spoken dialects and syntax (i.e., using different rules and principles to govern the sentence structure) are used by Chinese people in Canada and Taiwan. The extant DQOL in Chinese language was developed based on the dialect of Cantonese [9], where people in Taiwan use Mandarin Chinese. To address this issue, we included all items of the extant Chinese version form Canada [9], but explicitly modified syntax of individual item. For example, we replaced an item "" of the extant Chinese version from Canada by the item "" for Taiwanese. After item modification and replacement, we translated our Taiwanese version of the DQOL back to an English version [25] and compared the semantics of the translated English version to the original English version. We also invited seven diabetes patients (four males and three males; age range 60–80 years) from the same hospital and applied cognitive debriefing tests to assess the level of comprehension and cognitive equivalence of the items. The finding from cognitive debriefing tests suggests a minor revision in the wordings for some items.

This Taiwanese version includes the same items as those in a Chinese version developed in Canada [9]. Compared to the original DQOL, for satisfaction domain, we dropped one item asking about sexual life (How satisfied are you with your sex life?), and replaced it with a new item for diabetes control (How satisfied are you with your control over your diabetes?). For impact domain, we dropped two items asking about interference with sexual life (How often does your diabetes interfere with your sex life?) and insulin reactions (How often do you hide from others the fact that you are having an insulin reaction?). We replaced them with two new items on eating out (How often does your diabetes interfere with your eating out?) and traveling/vacation (How often do you avoid a vacation or trip because of your diabetes?). For worry domain, consistent with a Chinese version from Canada we dropped seven items asking about social/vocational worry associated with marriage, children, education, job, and insurance because these items are appropriate for younger adults. We, however, added three items relevant to worry about requiring insulin in the future (How often do you worry about requiring insulin in the future?), death (How often do you worry about death due to diabetes?), and eating food (How often do you worry about eating the wrong food?). The resulting version of the DQOL consisted of 42 items measuring three domains: 15 items for the satisfaction with treatment domain, 20 items for the impact of treatment domain, and 7 items for the worry about future effect of diabetes domain. Our factor analysis suggests a goodness-of-fit for the factorial structures of the translated DQOL [24]. The detailed process of developing a Taiwanese version of the DQOL has been described in our previous study [24].

All items are scored on a five-point Likert scale, ranging from 1 (very satisfied) to 5 (very dissatisfied) in satisfaction domain, and from 1 (never) to 5 (all the time) in impact and worry domains. Domain scores were calculated by summing responses of all items in the corresponding domains, and lineally transforming them to a 1–100 scale with higher scores representing poorer HRQOL. A summary score (overall HRQOL) is further derived by summing three domain scores and lineally transforming to a 1–100 scale.

### Other HRQOL measures: the D-39S and the RAND-12

We collected other HRQOL measures to validate the DQOL, including the D-39S and the RAND-12. The D-39S is a short-form (23 items) of the D-39, which is a diabetes-specific HRQOL instrument designed for patients with type-1 and type-2 diabetes [10]. The D-39 has been translated to Chinese language by our research team and demonstrates good psychometric properties [7]. We shortened the D-39 using the Ant Colony Algorithm and structural equation modeling, which specifically retained items showing best correlation with clinical variables and goodness-of-fit for the construct of interest [28]. The D-39S covers the same domains as the D-39: energy and mobility, diabetes control, anxiety and worry, social burden, and sexual functioning. Items are administered using seven response categories with score ranging from 1 (not affected at all) to 7 (extremely affected). Domain scores are calculated by summing all items in the same domain, and linearly transformed them to 1–100, with high scores representing poor HRQOL.

The RAND-12, a generic HRQOL instrument, is a short-form of the RAND-36 [29]. The RAND-12 uses 12 items to capture two underlying constructs: physical and mental health. We calculated two summary scores, a physical health composite (PHC) and mental health composite (MHC), which are norm-based standardized scores with a mean 50 and a standard deviation 10. Higher scores in PHC and MHC represent better HRQOL. We used the RAND-12 PHC and MHC instead of the SF-12 physical component score (PCS) and mental component score (MCS) because evidence suggests that the SF-12 might be less sensitive to detect important difference in HRQOL between the known groups [30].

### Psychometric analyses for the DQOL

Psychometric properties of the DQOL were examined using internal consistency (reliability), convergent/discriminant validity, and known-groups validity.

Internal consistency of each domain was estimated using Cronbach's alpha coefficient. An alpha of ≥ 0.7 is considered to be acceptable for the purpose of group comparisons [31]. Convergent and discriminant validity was assessed through a multi-trait multi-method (MTMM) which compares Pearson's correlation coefficients among domains of the DQOL with the D-39S and the RAND-12. As described in the Introduction, because the DQOL essentially measures satisfaction and impact of diabetes treatment, whereas the D-39S measures physical functioning and psychological well-being associated with diabetes, we hypothesized that the two DQOL domains (satisfaction with treatment and impact of treatment) would be more strongly associated with physical domains of the D-39S (diabetes control and energy and mobility) compared to with psychosocial domains of the D-39S (social burden, anxiety and worry, and sexual functioning). We also hypothesized that the worry domain of the DQOL which focuses more on physical aspects (such as worry about complication, change of physical appearance and death) would be strongly associated with physical domains of the D-39S (diabetes control and energy and mobility) compared to with psychosocial domains of the D-39S (social burden, anxiety and worry, and sexual functioning). With respect to the association between the DQOL and the RAND-12, we assumed that the DQOL domains would be more strongly associated with PHC compared to with MHC. A magnitude of Pearson's correlation coefficient 0–0.39, 0.4–0.69, and ≥ 0.7 is classified as weak, moderate, and strong, respectively [31].

Known-groups validity of the DQOL was examined by the extent to which the DQOL can discriminate between clinically well-defined patient groups, including laboratory diagnosis and diabetic complication groups. Laboratory diagnosis known groups are for those patients whose values of laboratory measures were below vs. above the accepted cut-off points: 110 mg/dL for FPG, 140 mg/dL for 2 h PPG, and 7.0% for HbA1c [32]. Diabetes complication known groups are for patients who were diagnosed with vs. without complications of retinopathy, neuropathy, diabetic foot diseases, cardiovascular, and cerebrovascular diseases, respectively. We calculated Cohen's effect size (ES) to indicate the magnitude of known-groups validity (unit: standard deviation [SD]) [31], defined as the differences in domain scores between known groups (e.g., HbA1c below vs. above 7.0%) divided by the pooled standard deviation of both groups. A magnitude of effect size < 0.2 SD, 0.2–0.49 SD, 0.5–0.79 SD, and ≥ 0.8 SD is classified as negligible, small, moderate, and large, respectively. We hypothesized that, compared to the RAND-12 the DQOL and the D-39S would discriminate better between laboratory known groups (with a ES ≥ 0.2) because these two instruments center on burden of diabetes treatment and symptoms of glycemic control. We also hypothesized that, compared to the RAND-12 the DQOL and the D-39S would discriminate better between the known groups of retinopathy, neuropathy, and diabetic foot complications rather than the known groups of cardiovascular and cerebrovascular complications. This is because these three complications are closely associated with diabetes treatment and control, and their impact could be directly captured by the domains included in both diabetes-specific instruments.

We also compared scores of the DQOL domains by treatment regimens, including 1) lifestyle modification alone or lifestyle modification plus oral agent (L/LO) and 2) lifestyle modification plus insulin or lifestyle modification plus oral agent and insulin (LI/LOI). We hypothesized that patients who were treated with L/LO regimen would demonstrate better HRQOL compared to patients who were treated with LI/LOI regimen.

### Establishment of minimally important difference

We used a cross-sectional method to determine MID which compares HRQOL scores in patients who were classified by level of health-relevant criteria [3,19]. Because there is no consensus on the superiority of a anchor- vs. distribution-based approach to determine MID (also see Introduction), we specifically combined the findings using a anchor-based approach (differences between health distinguishable groups) with a distribution-based approach [21,23].

Three-single items measuring patient's self-reported diabetes severity, general health status, and global quality of life were considered as anchors. Items of diabetes severity and global quality of life were rated by a seven categories, with scores ranging from 1 to 7 (from most severe/very dissatisfied to least severe/very satisfied). The item of general health status was rated by a five categories, with score ranging from 1 to 5 (poor, fair, good, very good, and excellent). We estimated differences in average HRQOL scores across adjacent categories of a specific anchor [21,23,33]. We considered the MID to be the difference in average scores corresponding to the effect size between 0.2 and 0.5 [23,34].

For a distribution-based approach, we estimated a standard error of measurement (SEM) which accounts for reliability of the DQOL and standard deviation of patients under the investigation. SEM was estimated by a standard deviation of the DQOL scores multiplied by a square root of one minus internal consistency of the DQOL scores. Based on evidence supported by Wyrwich and colleagues, we adopted a one-SEM criterion to reflect MID [35,36]. We finally used the findings derived from three anchors and a SEM to generate a range of MID values for individual DQOL domain.

In this study, all of the analyses were performed using the STATA 9.02 [37].

## Results

### Patient characteristics

Table 1 shows patients' characteristics (N = 337). Briefly, mean age was 61.6 years (SD: 10.9) and 51% were male. For laboratory indicators, mean FPG was 151 mg/dL (SD: 48), mean 2 h PPG was 204 mg/dL (SD: 78), and mean HbA1c was 7.9% (SD: 2.0). For diabetes complications, 16% had retinopathy, 14% had cardiovascular disease, 13% had diabetic foot disorder, 13% had neuropathy, and 5% had cerebrovascular disease. The majority of the subjects (88%) were treated with lifestyle modification or lifestyle modification plus oral agent.

**Table 1:** Patients characteristics (N = 337)

	Mean (SD) or %
**Demographic variables**	
Age in years, mean (SD)	61.6 (10.9)
<55, %	25.2
55–59.9,%	14.0
60–64.9	19.3
65–69.9	18.4
70–74.9	10.4
≥ 75	12.8
Gender, %	
Male	50.7
Female	49.3
Education, %	
No formal education	7.1
Primary and junior high schools	52.5
Senior high school	21.4
College	17.8
Graduate	1.2
Employment status, %	
Yes	40.4
No	59.6
	
**Laboratory tests**	
Fasting plasma glucose (FPG), mg/dL	150.7 (47.8)
2-hour postprandial plasma glucose (2 h PPG), mg/dL	204.0 (78.0)
Hemoglobin A1c (HbA1c), %	7.9 (2.0)
	
**Diabetes complications**	
Retinopathy, %	16.1
Neuropathy, %	12.5
Diabetic foot complications, %	12.5
Cardiovascular complications, %	14.0
Cerebrovascular complications, %	5.3
	
**Number of comorbid conditions, mean (SD)**	1.8 (1.2)
**Duration of DM in years, mean (SD)**	9.2 (6.3)
**Type of treatment, %**	
Lifestyle modification alone or lifestyle modification plus oral agent	87.9
Lifestyle modification plus insulin or lifestyle modification plus oral agent and insulin	12.1
**Type of diabetes^†^, %**	
Type-1 (age<30 years old and BMI<23)	1.2
Type-2 (either age ≥ 30 years old or BMI ≥ 23, or both)	98.8

† Classification of type of diabetes [49,50]

### Internal consistency

Internal consistency was ≥ 0.7 for all domains of the DQOL (0.90, 0.89, and 0.83 for impact, satisfaction, and worry domains, respectively).

### Convergent/discriminant validity

Table 2 shows convergent and discriminant validity of the DQOL against the D-39S and the RAND-12. In general, domain scores of the DQOL were moderately correlated with the D-39S (except sexual functioning) and the RAND-12 PHC and MHC, with Pearson's correlation coefficients ≥ 0.4. Magnitudes in the correlations of all DQOL domains with the diabetes control and energy/mobility of the D-39S were slightly larger than with the other D-39S domains (social burden, anxiety and worry, and sexual functioning). For example, Pearson's correlation coefficients of the satisfaction domain of the DQOL with diabetes control and energy/mobility of the D-39S were all 0.57, which were larger than with the other D-39 domains (0.30 through 0.46). Magnitudes in the correlations of all DQOL domains with the RAND-12 PHC were slightly larger than with the RAND-12 MHC.

**Table 2:** Convergent/discriminant validity the DQOL†

	DQOL SAT‡	DQOL IMP‡	DQOL WOR‡	DQOL ALL‡
**D-39**				
Diabetes control	-0.57§	-0.66	-0.54	-0.70
Energy and mobility	-0.57	-0.68	-0.54	-0.71
Social burden	-0.46	-0.65	-0.47	-0.64
Anxiety and worry	-0.51	-0.59	-0.46	-0.62
Sexual functioning	-0.30	-0.37	-0.29	-0.38
**RAND-12**				
PHC#	-0.53	-0.72	-0.57	-0.73
MHC#	-0.50	-0.68	-0.53	-0.69

† Values in the cells are Pearson's correlation coefficients: weak (0–0.39), moderate (0.40–0.69), and strong (≥ 0.7)

‡ SAT: satisfaction; IMP: impact; WOR: worry; ALL: overall

§ Negative value in Pearson's correlation coefficients is due to better HRQOL indicated by lower scores for the DQOL and the D-39S and higher scores for the RAND-12

# PHC: physical health composite; MHC: mental health composite

### Known-groups validity

Table 3 shows the known-groups validity tested using laboratory indicators and diabetes complications. After adjusting for age, gender, education background, and diabetes duration, the impact, worry, and overall HRQOL domains of the DQOL demonstrated discernible discriminative ability for 2 h PPG groups (effect sizes in score difference ≥ 0.2), but the satisfaction domain did not (effect size <0.2). For HbA1c groups, the satisfaction, worry, and overall HRQOL domains of the DQOL demonstrated discernable discriminative ability, but the impact domain did not. Discriminative ability of the DQOL and the D-39S by 2 h PPG and HbA1c known groups was compromised, depending on specific domains. Compared to the RAND-12, both diabetes-specific HRQOL instruments showed slightly better discrimination by using laboratory indicators. No specific domains of the DQOL, the D-39S, and the RAND-12 showed discernible discriminative ability for FPG groups.

**Table 3:** Known-groups validity the DQOL†

	DQOL SAT‡	DQOL IMP‡	DQOL WOR‡	DQOL ALL‡	D39 DC‡	D39 EM‡	D39 SB‡	D39 AW‡	D39 SF‡	RAND-12 PHC‡	RAND-12 MHC‡
**Laboratory indicators**											
Fasting plasma glucose	0.04§	0.06	0.12	0.08	0.08	0.07	0.05	0	-0.19	0.01	0.01
2-hour postprandial plasma glucose	0.17	0.24	0.34	0.28	0.39	0.40	0.26	0.21	0.43	0.34	0.20
Hemoglobin A1c	0.23	0.17	0.22	0.23	0.19	0.20	0.20	0.14	-0.04	0.15	0.11

**Diabetes complications**											
Retinopathy	0.10	0.42	0.31	0.35	0.36	0.48	0.41	0.47	0.31	0.30	0.07
Neuropathy	0.24	0.44	0.46	0.45	0.49	0.57	0.64	0.43	0.44	0.49	0.24
Diabetic foot complications	0.08	0.39	0.34	0.33	0.45	0.60	0.59	0.47	0.40	0.50	0.23
Cardiovascular complications	-0.06	0.20	0.14	0.13	0.21	0.32	0.15	0.18	0.32	0.27	0.13
Cerebrovascular complications	-0.09	0.08	0.04	0.03	0.12	0.30	0.41	0.31	-0.09	0.74	0.60

† Covariate adjustment: age, gender, education background, and duration

‡ SAT: satisfaction; IMP: impact; WOR: worry; ALL: overall; DC: diabetes control; EM: energy and mobility; SB: social burden; AW: anxiety and worry; SF: sexual functioning; PHC: physical health composite; MHC: mental health composite

§ Values in the cells are effect size: negligible (< 0.2), small (0.2–0.49), moderate (0.5–0.79), and large (≥ 0.8)

For the diabetes complication known groups, after adjusting for age, gender, education background, and diabetes duration, the impact, worry, and overall HRQOL domains of the DQOL demonstrated better discrimination than the satisfaction domain. This is especially evident for the known groups of retinopathy, neuropathy, and diabetic foot complications. Taking neuropathy as an example, the effect sizes in score differences of the impact, worry, and overall HRQOL domains were 0.44, 0.46, and 0.45, respectively, which were larger than the satisfaction domain (0.24). The discriminative ability of the DQOL and the D-39S by the known groups of retinopathy, neuropathy, and diabetic foot complications was compromised. Compared to the RAND-12, both diabetes-specific HRQOL instruments showed slightly better discrimination by laboratory indicators. By contrast, compared to the RAND-12 the discriminative ability of the DQOL and the D-39S was compromised by cardiovascular complication known groups, but less satisfied by cerebrovascular complication known groups.

### Treatment effect

**Figure 1:**
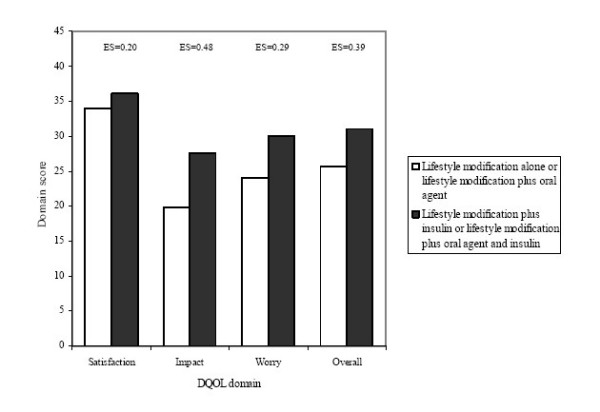
**Relationship between DQOL score and type of treatment**. ES: effect size, which is the difference in HRQOL scores (unit: SD) of the DQOL between two treatment categories.

Figure 1 shows associations of HRQOL and types of diabetes treatments – 1) lifestyle modification alone or lifestyle modification plus oral agent (L/LO) and 2) lifestyle modification plus insulin or lifestyle modification plus oral agent and insulin (LI/LOI). As we hypothesized, after adjusting for age, gender, education, and diabetes duration patients treated by LI/LOI regimen were associated with more impaired HRQOL in all domains than patients treated by L/LO regimen. The effect sizes in the score differences between two regimens for satisfaction, impact, worry, and overall HRQOL domains were all above 0.2 (i.e., 0.20, 0.48, 0.29, and 0.39, respectively), indicating clinically important difference.

### Minimally important differences

Table 4 shows MID for individual DQOL domain estimated using the anchor- and distribution-based approaches. Because case numbers were small for categories 1–3 in the self-reported diabetes severity and global quality of life anchors, these three categories were collapsed. For the self-reported diabetes severity anchor, MIDs (estimated by averaging differences in values between adjacent categories with corresponding effect size 0.2–0.5) were 2.6, 4.1, 6.5, and 3.1 points for the domains of satisfaction, impact, worry, and overall HRQOL, respectively. For the general health status anchor, the estimated MIDs were 4.9, 3.6, 5.6, and 3.7 points for the domains of satisfaction, impact, worry, and overall HRQOL, respectively. For the global quality of life anchor, the estimated MIDs were 3.0, 4.5, 7.0, and 4.1 points for the domains of satisfaction, impact, worry, and overall HRQOL, respectively. SEM of the distribution-based approach shows the MIDs were 3.7, 4.6, 8.0, and 3.0 points for the domains of satisfaction, impact, worry, and overall HRQOL, respectively.

**Table 4:** Minimally important differences of the DQOL

	Anchor 1: Self-reported diabetes severity				Anchor 2: General health status				Anchor 3: global quality of life				SEM
	
DQOL	Category	Mean	Difference in mean†	MID§	Category	Mean	Difference in mean	MID	Category	Mean	Difference in mean	MID	
SAT#	1–3	39.42	-	2.62	1	44.33	-	4.90	1–3	42.21	-	2.99	3.71
	4	36.33	3.12‡		2	36.58	13.24		4	36.36	5.87		
	5	33.66	2.32‡		3	32.00	5.11‡		5	33.23	2.99‡		
	6	31.28	2.41‡		4	26.26	4.68‡		6	32.05	1.38		
	7	24.52	7.54		5	14.38	8.48		7	23.24	8.50		

IMP#	1–3	32.17	-	4.06	1	35.58	-	3.60	1–3	28.82	-	4.52	4.58
	4	22.17	10.26		2	22.17	3.59‡		4	22.58	5.70‡		
	5	19.42	2.88‡		3	19.05	7.94		5	20.30	2.51		
	6	15.36	4.01‡		4	10.61	3.64‡		6	15.99	4.95‡		
	7	10.28	5.29‡		5	7.81	13.74		7	12.33	2.91‡		

WOR#	1–3	33.93	-	6.53	1	32.50	-	5.62	1–3	26.74	-	6.97	7.97
	4	26.90	7.28‡		2	27.34	4.58‡		4	29.41	-3.26		
	5	25.46	1.46		3	25.98	14.02		5	23.19	6.66‡		
	6	19.78	5.79‡		4	10.63	1.89		6	21.93	2.30		
	7	10.02	10.93		5	9.38	6.67‡		7	12.84	7.27‡		

ALL#	1–3	35.05	-	3.09	1	38.19	-	3.72	1–3	33.25	-	4.13	3.02
	4	28.02	7.22		2	28.18	7.21		4	28.64	4.27‡		
	5	25.51	2.44‡		3	24.83	7.94		5	25.40	3.37‡		
	6	21.78	3.73‡		4	16.20	3.72‡		6	22.72	3.23‡		
	7	15.32	7.03		5	10.42	10.68		7	16.31	5.63‡		

† Covariate adjustment: age, gender, education background, and duration

‡ Differences in mean HRQOL scores across adjacent categories with corresponding effect size 0.2–0.5

§ MID: minimally important difference, which is the average of the differences in mean HRQOL scores across adjacent categories with effect size 0.2–0.5

# SAT: satisfaction; IMP: impact; WOR: worry; ALL: overall

Combining the findings from anchor- and distribution-based approaches, the range of MIDs were 3–5 points for the satisfaction domain, 4–5 points for the impact domain, 6–8 points for the worry domain, and 3–4 points for the overall HRQOL.

## Discussion

Although the DQOL has been widely used in many studies [25-27], rigorous psychometric assessments for a Chinese language version are still limited. In this study, we tested psychometric properties of the DQOL in Chinese language for diabetes patients treated in Taiwan. Our version was harmonized with a previous version developed for Chinese people in Canada [9], which may facilitate the use in Chinese populations worldwide.

Our findings indicate that scores of the DQOL were moderately correlated with the D-39S (except sexual functioning) and the RAND-12. Magnitudes in the correlations of all DQOL domains with the physical relevant domains of the D-39S (diabetes control and energy/mobility) were slightly larger than with other domains of the D-39S (social burden, anxiety and worry, and sexual functioning). Additionally, magnitudes in the correlations of all DQOL domains with the RAND-12 PHC were slightly larger than with MHC. A DQOL validation study by Yildirim and colleagues reported that domains scores of the DQOL were more strongly correlated with the physical domains (e.g., mobility, vision, hearing, breathing, and so on) of the 15D (a generic HRQOL measure) than with the psychological domains (e.g., mental function, depression, distress, and so on) [38]. Another DQOL validation study by Jacobson and colleagues also reported that domains scores of the DQOL were more strongly correlated with the physical domains (e.g., role physical functioning and general health) of the SF-36 than with the psychosocial domains (e.g., social functioning) [25]. Taken together, these findings might suggest that the concepts captured by the DQOL, such as satisfaction with treatment, impact of treatment, and worry about future diabetes effects (complications, change of physical appearance and death), are more physical than psychosocial relevant. Therefore, the HRQOL constructs included in the DQOL and the D-39S are not completely equivalent.

For known-groups validity, we found that, compared to the RAND-12 both diabetes-specific HRQOL instruments demonstrated slightly better discrimination by known groups of 2 h PPG and HbA1c. Additionally, compared to the RAND-12 both diabetes HRQOL instruments (DQOL and D-39S) discriminated better between the known groups of retinopathy, neuropathy, and diabetic foot complications than the known groups of cardiovascular and cerebrovascular complications. This finding may be in part due to the fact that the indicators of 2 h PPG, HbA1c, retinopathy, neuropathy, and diabetic foot complications are closely associated with diabetes treatment and diabetes control, and their impact might be directly captured by the domains included in both diabetes-specific instruments. In contrast to retinopathy, neuropathy, and diabetic foot complications, the impact of cardiovascular and cerebrovascular complications (especially for stroke as an example) on daily functioning is more significant and might not be directly attributed to glycemic control, which could be better captured by the RAND-12. This finding suggests that it might be an ideal approach to use diabetes-specific HRQOL instruments combined with generic HRQOL instruments to fully measure HRQOL burden for diabetes patients [7,39,40].

Our study extends conventional methods used to validate diabetes HRQOL instruments. Previous studies have often used HbA1c as a glycemic control indicator to validate HRQOL instruments. However, evidence is mixed regarding the association between HbA1c and HRQOL [1,41-43]. Our results suggest that 2 h PPG may be a more sensitive laboratory indicator to validate HRQOL in patients with diabetes. Rather than averaging blood glucose levels from the preceding 2–3 months, 2 h PPG captures short term fluctuations in metabolic control. Epidemiologic studies have reported that patients with normal HbA1c, but abnormal 2 h PPG, are more prone to postprandial hyperglycemia, leading to substantially an increased risk of death from macrovascular diseases [13,44]. In most cases, PPG levels increase before and faster than FPG [45]. The usefulness of 2 h PPG over HbA1c and FPG has been demonstrated in our previous study to test validity of diabetes HRQOL measures using the D-39 [7].

A previous DQOL study suggests that patients with diabetes complications tended to report more impaired DQOL scores compared to their counterparts [9]. In this study, we support the value of using several individual complications as known groups to validate diabetes HRQOL instruments. We found that the effect sizes in the impact, worry, and overall HRQOL domains were greater than 0.2 for the known groups of retinopathy, neuropathy, and diabetic foot complications, suggesting clinically meaningful difference. Interestingly, the effect sizes in HRQOL scores between levels of laboratory indicators were generally smaller than for the presence and absence of diabetes complications. This may be due, in part, to the fact that clinical symptoms (e.g., hypoglycemia) and events (e.g., diabetic foot) are more evident to patients than laboratory abnormalities, leading to significant impairment on well-being.

Our study suggests that patients who received more intensive treatment (lifestyle modification plus insulin or lifestyle modification plus oral agent and insulin) was associated with the more impaired HRQOL in all domains compared to patients who received less intensive treatment (lifestyle modification alone or lifestyle modification plus oral agent). These comparisons were independent of the influence of age, gender, education, and diabetes duration. Johnson et al reported that, using the SF-12 patients on oral medication plus insulin had significantly lower physical and mental health than patients on oral medication, followed by lifestyle modification alone [30]. Similarly findings were also reported by Saito et al using the SF-36 [46]. However, some longitudinal studies reported that HRQOL was not significantly changed after patients taking insulin therapy [26,43]. This discrepancy may be due to the fact that some factors, e.g., increased patient education, family support, and decreased hyperglycemic symptoms, may offset the discomfort and problems related to insulin therapy.

To facilitate interpretation of the DQOL scores, we estimated the minimally important difference (MID). MID has been defined as the smallest difference in a HRQOL measure that is perceived by patients as being clinically meaningful [3,47]. Importantly, the choice of anchors might influence the MID estimation. Guyatt et al suggested that a useful anchor for MID should be interpretable and moderately correlated with target instruments [3,48]. Revicki et al recommended that anchors derived from patient's perspective should be given the most weight because they reflect the intuitive interpretation for the change in patient-reported outcomes [20]. In this study, three anchors we used (i.e., patient's self-reported diabetes severity, general health status, and global quality of life) were all based on patient's viewpoint. We specifically found that when the levels of external indicators indicated impairment, HRQOL measured by the DQOL showed impairment. Furthermore, these indicators were moderately correlated with the DOQL scores (Pearson's correlation coefficients > 0.4). As a result, we consider these indicators to be legitimate anchors and potentially be useful by other studies to interpret diabetes HRQOL measures.

Because neither anchor-based nor distribution-based approaches are superior to one another, we estimated MIDs based on several anchors and combined these estimates with a distribution-based estimate (i.e., standard error of measurement) [21-23]. We found that the range of MIDs for the DQOL were 3–5 points for satisfaction domain, 4–5 points for impact domain, 6–8 points for worry domain, and 3–4 points for overall HRQOL. The estimation of MIDs can be helpful for calculating sample size when HRQOL is used as an end point in clinical investigations. Interestingly, the findings from this and earlier studies [23] suggest that the combined use of anchor-based and distribution-based approaches tend to expand the range of MIDs compared to using either approach. The MID derived from the distribution-based approach is more likely to be on the opposite end of the MID range compared to the anchor-based approach. More studies using different anchor and distribution-based approaches need to be conducted to confirm these findings.

There are a number of limitations to this study. First, the generalizability of our results may be limited because our samples were collected from a single center in Taiwan. Second, although the DQOL was designed for measuring patients with type-1 and type-2 diabetes, only 4 patients in our sample had type-1 diabetes. Therefore, the psychometric properties of DQOL are based largely on type-2 diabetes. Further studies are needed to replicate our finding in patients with type-1 diabetes. Third, we estimated MIDs using a cross-sectional rather than a longitudinal design. We calculated differences in average HRQOL scores across adjacent categories of an anchor for MID estimations [21,23,33]. However, the resulting differences between adjacent groups by a cross-sectional design may not accurately reflect longitudinal changes within the same group. This latter method is known as the minimally important change (MIC) or responsiveness [3,19]. A longitudinal design would be preferable approach to examine changes in HRQOL.

## Conclusion

There is a great need to develop and validate diabetes HRQOL instruments for Chinese populations. In this study, we validated a Taiwanese version of the DQOL in Chinese language for diabetes patients in Taiwan. We used different psychometric methods together with different laboratory indicators and diabetes complications to validate the DQOL. In addition to providing a useful questionnaire, we also used a combined anchor-based and distribution-based method to interpret the DQOL scores. Further evaluation and improvement are indicated, especially to estimate responsiveness.

## Competing interests

The authors declare that they have no competing interests.

## Authors' contributions

IH, JL, and CH conceived the study and its design. MW and CH collected the data in Taiwan. IH, JH, and WL analyzed the data. IH, JL, and CH interpreted the data. IH drafted the manuscript. IH, JL, AW, MW, WL, and CH revised critically for important intellectual content.
